# Recall and Self-Relevance of Emotional Words Predict Subjective Self-Evaluation of Cognition in Patients with MTLE with or without Depressive Symptoms

**DOI:** 10.3390/brainsci11111402

**Published:** 2021-10-24

**Authors:** Lidija Preglej, Ksenija Marinkovic, Hrvoje Hećimović

**Affiliations:** 1 Language and Cognitive Neuroscience, Interdisciplinary Scientific Postgraduate Program, University of Zagreb, 10000 Zagreb, Croatia; 2 Department of Psychology, San Diego State University, San Diego, CA 92182, USA; kmarinkovic@sdsu.edu; 3 Department of Radiology, University of California at San Diego, San Diego, CA 92093, USA; 4 Department of Nursing, University North, 42000 Varaždin, Croatia; hrvoje.hecimovic@gmail.com; 5 Neuro Center, 10000 Zagreb, Croatia

**Keywords:** mesial temporal lobe epilepsy, cognition, self relevance task, depression, BDI

## Abstract

We examined whether word processing is associated with subjective self-evaluation of cognition in patients with mesial temporal lobe epilepsy (MTLE) as a function of their depressive symptoms. MTLE patients with (MTLE +d, *N* = 28) or without (MTLE -d, *N* = 11) depression were compared to pair-matched healthy control participants on free recall and self-relevance ratings of emotionally valenced words. Correlation and hierarchical analyses were conducted to investigate whether the subjective self-evaluation of cognition in MTLE patients is predicted by the negative emotional bias reflected in task performance. MTLE +d patients endorsed as self-relevant fewer positive words and more negative words than the MTLE -d patients and healthy participants. They also self-evaluated their cognition poorer than the MTLE -d patients. Analyses indicated that recall and self-endorsement of emotional words predicted both self-evaluation of cognition as well as epilepsy duration. Our findings indicate that negative self-relevance emotional bias is observed in MTLE patients and is predictive of subjective self-evaluation of cognition. Application of brief behavioral tasks probing emotional functions could be valuable for clinical research and practice in the patients with MTLE.

## Highlights

1. Depression specific self-relevance bias is observed in MTLE patients with depressive symptoms.
2. Behavioral variables are predictors of subjective self-evaluation of cognition.
3. Epilepsy duration is a predictor of subjective self-evaluation of cognition.

## 1. Introduction

In clinical practice, patients with epilepsy frequently report cognitive difficulties, particularly in relation to the functions that rely on memory [1,2,3,4]. Such complaints are not surprising in patients with mesial temporal lobe epilepsy (MTLE) and are more prevalent in participants with hippocampal lesions [5,6,7]. However, self-evaluated cognitive difficulties are not always detected by neuropsychological evaluation [1,4,8,9]. Why it is the case is still an interesting and open question.

### 1.1. The Presence of Depressive Symptoms

One factor which may prevent the objective confirmation of self-evaluated cognitive difficulties in patients with MTLE could be related to present depressive symptoms in patients with MTLE. Studies on patients with epilepsy [3,8,9,10] and other disorders [9,11] suggest that subjective self-evaluated cognitive difficulties in the absence of objective cognitive deficits may be associated with the current presence of depressive symptoms detected by the Beck Depression Inventory (BDI). Importantly, earlier studies established a strong connection between the MTLE and depression [12,13,14,15], and negative impact of depression comorbidity on subjective self-evaluation of cognition was also found [16]. In terms of clinical variables related to depression, higher levels of depressive symptoms are suggested to be related to a later age of seizure onset [17], longer epilepsy duration [18,19], higher seizure frequency [20] and antiseizure medication polytherapy [21]. In addition, poorer subjective self-evaluation of cognition has been associated with longer epilepsy duration and higher seizure frequency [22,23]. Therefore, we examined whether the clinical variables previously shown to be relevant to depression could help predict the subjective self-evaluation of cognition in patients with MTLE. As described below, results of the present study emphasize the importance of recognizing depression in participants with MTLE.

Behavioral paradigms have been shown to be sensitive to depressive symptoms. Non-epilepsy participants with depression (clinical and subclinical) manifested cognitive vulnerability to depression [24,25] indicated by emotional depression-specific cognitive bias [26,27,28,29]. The emotional modification of a free recall task has been proven as sensitive to emotional depression-specific memory bias [29,30,31,32,33]. The emotional memory bias towards emotionally negative depression-specific stimuli [27,28,29] or/and away from emotionally positive stimuli [26,29,34] was shown in non-epilepsy participants with depression (both subclinical and clinical). Similar memory bias was shown in MTLE patients with depressive symptoms [35]. Solid neuroimaging evidence supports Beck’s theory of depression [36] according to which people with depressive symptoms tend to rate themselves through a depressive lens: less positively [26] and more negatively [37] than those without depressive symptoms. In the present study, we administered an emotional self-relevance task to the same participants who previously took part in emotional attentional and free recall tasks [35] to improve the sensitivity of our behavioral paradigm to depressive symptoms, potentially shown as a depression-specific cognitive bias.

### 1.2. The Presence of Depression-Specific Cognitive Bias without Current Depressive Symptoms

Another possible reason why self-reported cognitive difficulties are often not confirmed with objective assessment could be related to the underlying cognitive vulnerability to depression. Indeed, emotional depression-specific cognitive bias has been reported even in “non-epilepsy” participants without depressive symptoms [24,29,38,39,40], as well as in patients with MTLE without depressive symptoms (MTLE -d) [35,41,42]. Furthermore, similar findings were reported in “non-epilepsy” participants with depression in remission using a self-relevance task [26]. However, to the best of our knowledge, a self-relevance task has not been reported for patients with MTLE to address that matter. Thus, short behavioral tasks have potential clinical relevance as they could assist epileptologists and the associated clinical teams to recognize cognitive vulnerability to depression in patients with MTLE. Furthermore, it is possible that subjective self-reported cognitive difficulties in the absence of objective confirmation could be associated with cognitive vulnerability to depression in MTLE patients without depressive symptoms detected by the BDI (MTLE -d). Depression-specific patterns of processing emotionally valenced stimuli [26,27,28,29] have been shown to predict depression [43,44,45,46] in individuals without epilepsy. Therefore, to address these gaps, the present study examined behavioral indices of emotional depression-specific cognitive bias in patients with MTLE. These indicators of cognitive vulnerability to depression could be used as predictors of subjective self-evaluation of cognitive difficulties.

### 1.3. The Objective Memory Ability Assessment Method

One factor which may prevent the objective confirmation of subjective self-evaluated cognitive difficulties could be related to the manner of assessing MTLE patients’ cognitive abilities, which is often limited to standard test-based evaluations [1,2,3,4,8]. Indeed, the approach based exclusively on neuropsychological tests has been criticized by some authors [23,47] for its lack of ecological validity, meaning that such tests may not measure behaviors that are relevant to real-life settings. Therefore, behavioral tasks could potentially serve as an additional method for assessing cognitive abilities in patients with MTLE. 

The aims of this study were (a) to examine whether memory deficit and self-ratings of emotionally relevant stimuli are associated with the subjective self-evaluation of cognition in MTLE patients as a function of their depressive symptoms; and (b) to investigate whether the subjective ratings of cognitive difficulties are predicted by cognitive emotional bias and depression related clinical variables. We hypothesized that subjective self-evaluation of cognition is predicted primarily by depression measured with behavioral and clinical variables.

## 2. Methods

### 2.1. Participants 

A total of 78 participants were recruited for this study, as previously described in a companion publication [35]. The patient group comprised of 39 right-handed adult participants with pharmacoresistant MTLE (Table 1) who were recruited at the Tertiary Epilepsy Center at the University Hospital in Zagreb, Croatia. Their MTLE status was determined by a long-term video/EEG monitoring. Pharmacoresistance to antiepileptic drugs (AEDs) was defined according to the criteria proposed by the International League Against Epilepsy [48]. Exclusionary criteria were: evidence of any structural brain lesion as indicated by high resolution brain MRI scans with the exception of hippocampal sclerosis, and current use of any antidepressive medications. The control group included 39 healthy, right-handed participants with no history of neurological disorders or depressive symptoms (BDI<7). The two groups were matched on their age, sex and years of education in a pairwise manner (Table 1). All participants had normal or corrected-to-normal vision. The study was approved by the University Hospital Ethics Committee and a written informed consent was obtained from all participants. 

### 2.2. Experimental Procedure

All participants completed the BDI [49]. The MTLE group also completed the QOLIE-31 [50]. During the same visit, all participants took part in a free recall and an emotional variant of modified self-relevance ratings tasks.

#### 2.2.1. Inventories

##### Beck Depression Inventory—II (BDI-II)

The BDI score ≥ 15 was considered indicative of clinically relevant depressive symptoms [29,45,51]. None of the participants in the control group manifested depressive symptoms. Patients (*N* = 28) with a total BDI score < 15 were included in the group of MTLE participants without depression (MTLE -d), and participants with depressive symptoms (MTLE +d), *N* = 11 had total BDI scores ≥ 15 (Table 1).

##### Quality of Life in Epilepsy Inventory—31 (QOLIE-31)

In the same session, the MTLE patients completed the QOLIE-31 [50] which contains 31 questions providing self-assessment in seven domains: worries related to seizures, emotional well-being, energy/fatigue, cognitive functioning, medication side effects, social functioning, and the overall quality of life. Within the “cognitive functioning” domain, subsets of questions refer to self-ratings of memory problems, the ability to concentrate, and the problem-solving capacity. Higher scores reflect higher levels of the perceived quality of life in each domain. The cognitive score (QOLIE CS) was used to assess the subjective self-evaluation of cognition in patients with MTLE, as reported in previous studies [9,22].

#### 2.2.2. Experimental Behavioral Paradigm

The task of spatial cueing of attention with emotional modification was described in detail in the accompanying publication [35]. Briefly, participants were instructed to pay attention to words with the positive, neutral and negative valence that were individually presented on the left or right side of the screen. After each word, a target shaped as a dot appeared on one side of the screen and participants were asked to respond to the target location by pressing the relevant button (left/right) of the response box as fast as possible. Words and targets were presented on the left and right side with equal probability and in random order. An incidental, surprise free recall memory task [30,33] was administered immediately after the attentional task. Participants were asked to write down as many words as they could recall [35].

##### Self-Relevance Ratings Task

Participants [35] additionally completed a self-relevance ratings task in the same session that included attentional and memory tasks. A subset of 24 words from the attentional task with equiprobable emotional representation (the same number of words per category) was used to assess if these emotional words described the participants’ current experience and feelings. Participants were asked to evaluate each word for self-relevance by deciding whether it described himself/herself in the past two weeks (yes/no). The words were individually presented in a randomized order in the center of the computer screen until a response was given with either the left or right hand. Response mapping was counterbalanced across participants so that half of them were asked to press the right button for “yes” and the left button for “no” and vice versa. A practice block of 6 trials preceded the self-relevance task. The number of self-relevant words (SRW) endorsed for each emotional category was calculated for each subject.

##### Experimental Stimuli

A total of 48 words in the Croatian language were divided into positive, neutral and negative categories based on their emotional valence, with 16 words in each category [35]. These stimuli were chosen from a much larger set consisting of 602 words selected from the Dictionary of Croatian Synonyms [52]. Fifty independent judges rated emotional valence of the words on the Likert scale ranging from 0 (very negative) to 9 (very positive). The words selected into the three categories were highly representative of their respective emotional valence as confirmed by their ratings: negative (1.92 ± 0.21), neutral (5.00 ± 0.21), and positive (8.10 ± 0.20). There was no overlap among the word categories as positive and negative words were carefully selected to be equidistant from the neutral words with respect to their valence ratings. The three word lists differed in their emotional valence, [F(2,47) = 3558.85; *p* < 0.01], with the neutral word rated significantly different from positive (*t* (30) = 42.93; *p* < 0.01) and negative words (*t* (30) = 41.51; *p* < 0.01). 

All negative words were based on self-concepts related to depression (e.g., ‘sadness’) [31,36]. None of the words had any reference either to epilepsy symptoms or to other negative emotions (e.g., disgust, fear). Furthermore, the selection of negative (depressive) words was conducted based on an additional confirmatory assessment provided by patients with major depressive disorder (*N* = 20) according to self-relevance. All words on the three emotional word lists (positive, neutral, negative) were matched on length (7.73 ± 0.79 letters) and the frequency of use (0.001 ± 0.002 words per million). The three-word conditions were equated on the number of adjectives (14) and nouns (2). All words were additionally evaluated for their concreteness/abstractness by the 50 independent judges on a Likert scale ranging from 1—extremely concrete, to 8—extremely abstract. As expected, neutral words (2.66 ± 0.71) were rated as more concrete compared to positive (4.66 ± 0.94), (*t* (30) = 6.79; *p* < 0.01) and negative words (4.39 ± 0.78), (*t* (30) = 6.61; *p* < 0.01). 

### 2.3. Data Analysis

For the free recall task, the analyses were conducted on the total number of the correctly recalled words (RecWt) across participant groups. The variable “free-recall for positive words”, which was shown to be a behavioral indicator of emotional depression-specific memory bias in our previous work [35] was further tested in the correlation analysis. For the self-relevance ratings task, the number of selected self-relevant words (SRW) was analyzed for each emotional category across the three groups. The data were analyzed with mixed model ANCOVAs with age as a covariate with SPSS (Version 11.0, Chicago, IL, USA). 

Associations between the performance parameters, self-reports on depression and QOLIE scales, as well as the relevant clinical variables, were examined with the Pearson’s correlation coefficient that was calculated across both patient groups and corrected with the Benjamini–Hochberg correction to control for the false discovery rate [53]. Correlation analysis was conducted to examine the association of the behavioral performance variables and the relevant clinical variables on QOLIE CS. 

## 3. Results

### 3.1. Self-Evaluation of Cognition in Participants with MTLE 

Based on QOLIE CS scores, the two patient groups (MTLE -d, MTLE +d) differed in their subjective self-evaluations of cognition, (F(1,37) = 24.33; *p* < 0.01). MTLE +d patients rated their cognitive functions as being more impaired (254.2 ± 123.0) than the MTLE -d patients (440.9 ± 106).

### 3.2. Task Performance

#### 3.2.1. Overall Free Recall

**Figure 1:**
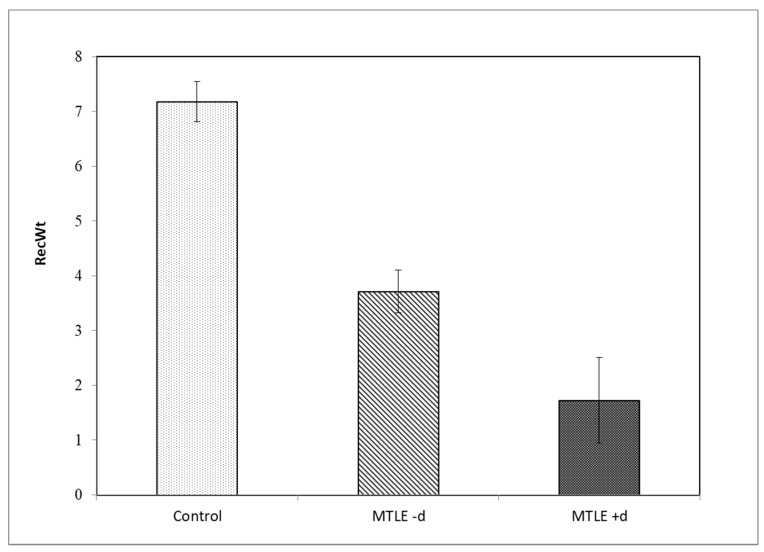
Total number of recalled words (RecWt) for the control, MTLE -d and MTLE +d groups. Error bars represent SEM values.

Control participants recalled more words in total than both MTLE -d and MTLE +d participants (Figure 1, Table 2).

A simple ANCOVA was used to examine differences in the total number of the recalled words (RecWt) between three groups (Control, MTLE -d, MTLE +d) with age as covariate. Results show a significant main effect of group (*F* (2, 74) = 13.05; *p* < 0.01), but no effect of covariate (age) (*F* (1, 74) = 1.45; ns). Post hoc analyses showed that control participants recalled more words than MTLE -d participants (*p* < 0.01) and MTLE +d (*p* < 0.01), while there was no significant difference between MTLE -d and MTLE +d (Figure 1).

The two MTLE groups did not differ in seizure focus laterality, number of antiepileptic drugs or demographic variables.

#### 3.2.2. Self-Relevance Ratings

**Figure 2:**
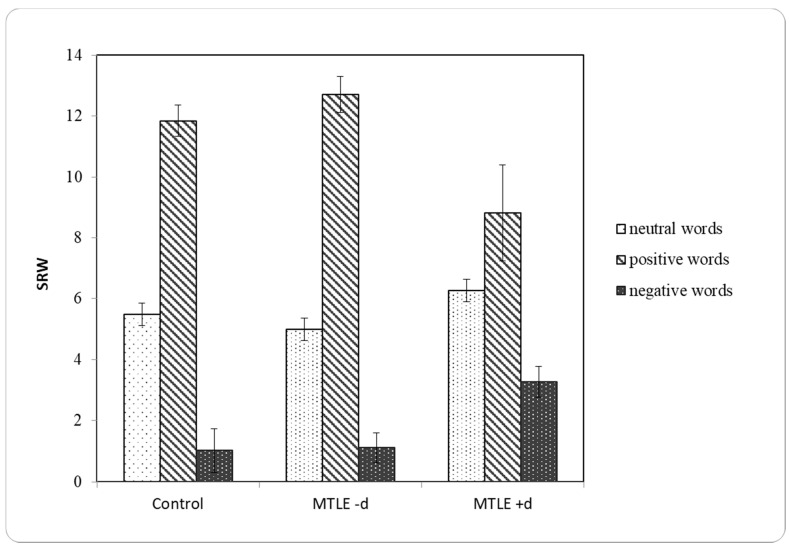
The number of endorsed self-relevant words (SRW) for each emotional valence word category for the control, MTLE -d and MTLE +d groups. Error bars represent SEM values.

MTLE +d patients endorsed as self-relevant fewer positive and more negative words in comparison to both the MTLE -d and control groups (Figure 2, Table 3).

A 3 × 3 mixed model ANCOVA with age as covariate was used to examine group differences (MTLE -d, MTLE +d, control) on the number of self-relevant words (SRWs) as a function of their emotional valence (positive, negative, neutral). 

Overall, a significant main effect of emotional word valence indicated that participants endorsed fewer negative words compared to positive (*p* < 0.001) and neutral (*p* < 0.001) (Table 3). They endorsed more positive than neutral words (*p* < 0.001). While the MTLE -d and control groups had similar self-relevance ratings (Table 3), MTLE +d participants endorsed more depressive and fewer positive words as relevant to their self-perception, as indicated by a group × valence interaction (Table 4). 

The three groups varied in the number of the positive words (SRWp) (*F* (2,76) = 4.820; *p* < 0.01). The patients with MTLE +d endorsed fewer positive words than the MTLE -d (*t*(36) = 2.86; *p* < 0.01) and controls (*t*(48) = 2.38; *p* < 0.05). The groups were different on the number of self-endorsed negative words (*F* (2,76) = 6.12; *p* < 0.01), with MTLE +d patients endorsing more negative words compared to both the MTLE -d (*t*(36) = 3.35; *p* < 0.01) and control group (*t* (48) = 2.92; *p* < 0.01) (Figure 2). Thus, the MTLE +d group showed a negative self-reference bias by endorsing fewer positive and more negative words than the MTLE -d and control groups.

### 3.3. Correlation Analysis

Subjective self-evaluation of cognition (QOLIE CS) was negatively associated with BDI, (*r* = −0.69; *p* < 0.01; confidence interval = −1 to −0.55).

RecWt correlated with QOLIE CS and with BDI (Table 5), indicating that the lower overall free recall was associated with higher BDI scores and with poorer subjective self-evaluation of cognition. RecWp and SRWp correlated negatively with BDI and positively with QOLIE CS, while SRWneg correlated positively with BDI (Table 5). 

Epilepsy duration (ED) correlated negatively with QOLIE CS and positively with BDI (Table 5). The age of seizure onset (ASO) and seizure frequency (SF) were not associated with QOLIE CS or BDI. Lower self-perception of cognition was expressed by older patients as indicated by a negative correlation between QOLIE CS and age (Table 5). 

### 3.4. Hierarchical Regression Analysis

Since a range of variables (RecWt, RecWp, SRWp, SRWneg, BDI, ED, age) were found to be associated with QOLIE CS, hierarchical regression analyses were conducted to examine their influence on QOLIE CS. In the first step, each variable was entered to examine its relation with QOLIE CS scores. The QOLIE CS score was predicted by BDI scores (β = −0.70; *p* < 0.01) and by behavioral variables: RecWt (β = 0.446; *p* < 0.01), RecWp (β = 0.38; *p* < 0.05), SRWp (β = 0.58; *p* < 0.01), SRWneg (β = −0.46; *p* < 0.01). Furthermore, QOLIE CS was predicted by ED (β = −0.62; *p* < 0.01) and by age (β = −0.42; *p* < 0.01).

## 4. Discussion

This study employed a combination of behavioral tasks, standardized inventories and clinical variables to characterize patterns of emotionally self-relevant memory deficits and subjective cognitive difficulties in patients with MTLE. We have found that behavioral tasks probing emotional functions could potentially serve as additional tools to evaluate both memory performance and depressive tendencies, with potential relevance to clinical practice. As expected, an overall memory deficit was confirmed in patients with MTLE in comparison to pair-matched healthy control participants. Patients with MTLE +d manifested a depression-specific processing bias as they endorsed as self-relevant fewer positive and more negative words than patients with MTLE -d and control participants. The patients with MTLE +d also evaluated their cognitive capacity as being more degraded than the MTLE -d group. The MTLE patients’ subjective self-evaluation of cognition was predicted by the total number of recalled words and by the number of self-rated positive words. Furthermore, both the number of recalled and endorsed as self-relevant positive words predicted the patients’ subjective self-evaluation of cognition.

We first examined whether memory deficit as well as self-relevance for depression-relevant stimuli can be observed in MTLE patients with and without depressive symptoms detected with the Beck Depression Inventory. We were particularly interested in applying behavioral tasks to address this question because the available evidence [35,42] indicates that tasks might be sufficiently sensitive to detect the cognitive vulnerability to depression in patients with MTLE, with or without depressive symptoms. Indeed, measures of free recall and self-ratings of words with emotional valence provided a deeper insight into the pattern of behavioral performance of our patients as a function of their depressive symptoms. Processing of positive words was of particular interest since it has been reported previously that these MTLE -d and MTLE +d participants differ on memory of positive words only [35]. Our MTLE -d patients recalled fewer positive words than control participants, even though they did not show current depressive symptoms on BDI [35]. Thus, behavioral tasks with depression-relevant stimuli may be potentially useful to recognize cognitive vulnerability to depression in MTLE patients even without depressive symptoms. More sensitive measures of emotional–cognitive bias used as indicators of depressive tendencies could benefit such patients as they could be offered remedial treatments. For instance, the patients with MTLE who completed cognitive behavioral therapy showed an improvement in depressive symptoms as reported by Orjuela-Rojas et al. [54]. The accompanying increase in the self-reported quality of life was comparable to those who were prescribed SSRI medications. Deficient processing of positive words observed in our MTLE +d patients, in comparison to both MTLE -d and healthy control participants, is consistent with the previously reported pattern in individuals with subclinical depression [26,55,56] and in those with natural and induced dysphoria [29]. In addition, the patients with MTLE +d in our study tended to show self-relevance bias towards negative stimuli, which is consistent with the reports of elevated memory for negative stimuli displayed by subclinically depressed individuals [28,29]. The observed bias away from positive and towards negative words in our MTLE +d patients was associated with their BDI scores in agreement with previous evidence in dysphoria [29], suggesting that the memory bias is impacted by the cognitive vulnerability to depression. Our behavioral findings are in line with the extensive evidence of greater depressive tendencies in patients with MTLE [12,13,14,15].

Next, we explored whether the described behavioral indices of patients with MTLE are associated with their subjective self-evaluation of cognition. MTLE +d patients exhibited a negative emotional self-relevance bias as they endorsed fewer positive and more of depression-relevant words (Table 3 and Table 4). They also showed poorer subjective self-evaluation of cognition. The patterns suggest that the self-attribution of positive descriptors decreases and the self-reported attribution of negative descriptors increases with higher depressive symptoms along with worsening of self-rated cognitive difficulties. Given the findings of cognitive emotional bias being predictors of depression [39,57], our findings indicate that behavioral variables could potentially serve as predictors of self-evaluation of cognition in patients with MTLE. 

An overall memory deficit observed in the MTLE patients lends support to other studies showing that the cognitive impairments documented in epilepsy patients are accompanied with subjective complaints about cognitive difficulties [1,2,3,4], including memory deficits [5,6,7]. In fact, the overall free recall scores of our patients with MTLE were negatively associated with their depressive symptoms and predicted their self-evaluation of cognition. Thus, our findings show that, in the context of objective memory difficulties displayed by our MTLE patients, their subjective cognitive complaints are strongly associated with depression, in agreement with other findings [3,8,9,10,16]. Our findings are aligned with Rayner et al. [2] who reported that both memory dysfunction and depression comorbidity predict subjective memory complaints in patients with MTLE. In contrast, in epilepsy patients with seizure foci outside the MTL area, only a history of depression predicted subjective complaints of memory dysfunction. Furthermore, Marino et al. [9] reported that subjective cognitive complaints are more related to mood than objective cognitive performance in patients with epilepsy, and also in those diagnosed with Parkinson’s disease.

Because the behavioral task revealed memory deficit in our MTLE patients, our study may contribute useful evidence from a methodological point of view. Using behavioral tasks to probe memory or other functions may have ecological validity [23,47], resulting in an increased likelihood of detecting functional deficits. On this view, the lack of ecological validity of some standardized tests [23,47] may be a reason for non-confirmed subjective self-evaluation of cognition in epilepsy patients’ evaluations [1,4,8,9]. Furthermore, the MTLE patients’ subjective self-evaluation of cognition was predicted by the total number of recalled words and the number of self-rated negative words. 

We have examined clinical variables as predictors of subjective self-evaluation of cognition and found that longer epilepsy duration predicts poorer subjective self-evaluation of cognition. Further, in line with the reports of subjective cognitive decline in healthy elderly adults [58,59], our older MTLE patients reported higher levels of subjective cognitive difficulties. However, the objective measures of their task performance were not associated with age. We found no difference in any of the behavioral variables as a function of seizure focus lateralization. It is possible that the usage of high frequency words and a relatively greater participation of patients with higher education levels mitigated such possible effects. 

The main limitation of our study is a relatively small sample of the patients with MTLE +d.

It is not an exception that epilepsy studies have small samples [42,60,61,62,63]. Future research could use a more extensive neuropsychological testing and include participants with extra temporal neocortical seizure foci to compare with these results. A longitudinal study design would be useful to better explore changes in cognitive dysfunction over time. In addition, the patients with psychiatric depression without epilepsy could provide better insight in relevant neural networks.

In summary, our results show that memory and self-relevance bias of emotionally valenced stimuli could be observed in the MTLE patients as a function of their cognitive vulnerability to depression. As suggested by tasks performance, cognitive depression tendencies have the strongest impact on subjective self-evaluation of cognition in participants with MTLE. Our findings indicate that the use of brief behavioral tasks can be potentially valuable additional tools for clinical research of cognition in subjects with epilepsy. They underscore the importance of early detection and treatment of vulnerability to depression in patients with MTLE.

## Figures and Tables

**Table 1:** Demographic and clinical characteristics of individuals with mesial temporal lobe epilepsy (MTLE) and healthy control participants.

	MTLE Group	MTLE -d	MTLE +d	Control Group
	*N* = 39			*N* = 39
Sex				
Male	11	8	3	11
Female	28	20	8	28
Age (y)	35 ± 11	33.39 ± 11.77	38.18 ± 8.86	34.54 ± 11.60
Education (y)	12.7 ± 2.2			12.7 ± 2.2
≤12	75.21%	21 (75.00%)	8 (72.72%)	75.21%
>12	24.79%	7 (25.00%)	3 (27.27)	24.79%
Total BDI score:	10.32 ± 8.18	5.85 ± 3.77	21.27 ± 5.00	3.82 ± 2.18
Seizure focus lateralization:				
Left	21 (54.85%)	12 (42.86%)	6 (54.5%)	
Right	18 (46.15%)	16 (57.14%)	5 (45.5%)	
Hippocampal sclerosis	1 (2.56%)	0	1 (9.1%)	
Number of seizures/month over past 6 months	1.54 ± 1.69	1.29 ± 1.36	2.18 ± 2.32	
Epilepsy duration (y)	15.46 ± 10.81	11.89 ± 9.02	24.55 ± 9.89	
Age at seizure onset (y)	19.10 ± 12.92	21.36 ± 12.57	13.36 ± 12.54	
AED therapy				
Monotherapy	56.40%	16 (57.14%)	6 (54.5%)	
Polytherapy	43.60%	12 (42.86%)	5 (45.5%)	

All values are given as means ± SD, total numbers, or percentages as indicated. Control participants were matched in a pairwise manner with MTLE patients on age, sex, years of education and handedness.

**Table 2:** Total number of recalled words (RecWt) for the control, MTLE -d and MTLE +d groups.

RecWt	Min	Max	M	SEM	SD
Control	1	23	7.18	0.719	4.489
MTLE -d	0	9	3.71	0.496	2.623
MTLE +d	0	5	1.73	0.506	1.679

Min = minimal number of recalled words; Max= maximum number of recalled words.

**Table 3:** Number of self-relevant words (SRW) for neutral (SRWneut), positive (SRWp) and negative (SREneg) emotional valence of words for the control, MTLE -d and MTLE +d groups.

Control	M	SEM	SD
SRWneg	1.03	0.332	2.071
SRWp	11.85	0.516	3.224
SRWneut	5.49	0.367	2.293
MTLE -d	M	SEM	SD
SRWneg	1.11	0.229	1.188
SRWp	12.70	0.592	3.074
SRWneut	5.00	0.389	2.019
MTLE +d	M	SEM	SD
SRWneg	3.27	0.854	2.832
SRWp	8.82	1.583	5.250
SRWneut	6.27	0.776	2.573

**Table 4:** Results of a 3 × 3 mixed model ANCOVA for self-relevance ratings of words for the control, MTLE -d and MTLE +d groups.

	*F*	Ss	*p*	η^2^
Within-subjects effects				
Emotional word valence	48.09	2/146	<0.001	0.397
Emotional word valence × age	7.16	2/146	<0.001	0.089
Emotional word valence × group	5.12	4/146	<0.001	0.123
Between-subjects effects				
Group	0.06	2/73	0.938	
Age	1.42	1/73	0.238	

**Table 5:** Correlation coefficients between inventory scores, behavioral and clinical variables for MTLE patients (*N* = 38).

	QOLIE CS	Confidence Interval 95%	BDI	Confidence Interval 95%
RecWt	0.43 **	0.047–0.591	−0.33 *	−0.455–0.130
RecWp	0.38 *	0.052–0.577	−0.27 ′	−0.311–0.204
SRWp	0.58 **	0.125–0.674	−0.46 **	−0.574–0.173
SRWneg	−0.46 **	−0.507–0.114	−0.54 **	−0.088–0.564
SF	−0.33	−0.514–0.109	0.21	−0.172–0.563
ED	−0.62 **	−0.645–(−0.172)	0.49 **	−0.216–0.359
ASO	0.17	−0.307–0.271	−0.25	−0.330–0.270
Age	−0.42 *	−0.642–(−0.136)	0.19	−0.265–0.301

Pearson’s correlation coefficient significance (2-tailed): ** *p* < 0.01, * *p* < 0.05, ′ *p* = 0.053.

## Data Availability

The medical data of patients were available only for the purpose of the research and taken from the internal data base of the hospital.
